# A bias‐free, automated planning tool for technique comparison in radiotherapy ‐ application to nasopharyngeal carcinoma treatments

**DOI:** 10.1120/jacmp.v15i1.4530

**Published:** 2014-01-06

**Authors:** Christopher Boylan, Carl Rowbottom

**Affiliations:** ^1^ The Christie NHS Foundation Trust Manchester UK; ^2^ Manchester Academic Health Science Centre (MAHSC) Faculty of Medical and Human Sciences, University of Manchester Manchester UK

**Keywords:** volumetric‐modulated arc therapy, intensity‐modulated radiotherapy, inverse optimization, treatment planning

## Abstract

In this study a novel, user‐independent automated planning technique was developed to objectively compare volumetric‐modulated arc therapy (VMAT) and intensity‐modulated radiotherapy (IMRT) for nasopharyngeal carcinoma planning, and to determine which technique offers a greater benefit for parotid‐sparing and dose escalation strategies. Ten patients were investigated, with a standard prescription of three dose levels to the target volumes (70, 63, and 56 Gy), using a simultaneous integrated boost in 33 fractions. The automated tool was used to investigate three planning strategies with both IMRT and VMAT: clinically acceptable plan creation, parotid dose sparing, and dose escalation. Clinically acceptable plans were achieved for all patients using both techniques. For parotid‐sparing, automated planning reduced the mean dose to a greater extent using VMAT rather than IMRT (17.0 Gy and 19.6 Gy, respectively, p<0.01). For dose escalation to the mean of the main clinical target volume, neither VMAT nor IMRT offered a significant benefit over the other. The OAR‐limiting prescriptions for VMAT ranged from 84‐98 Gy, compared to 76‐110 Gy for IMRT. Employing a user‐independent planning technique, it was possible to objectively compare VMAT and IMRT for nasopharyngeal carcinoma treatment strategies. VMAT offers a parotid‐sparing improvement, but no significant benefit was observed for dose escalation to the primary target.

PACS numbers: 87.55.D‐, 87.55.kd

## I. INTRODUCTION

Intensity‐modulated radiotherapy (IMRT) is an established treatment option for patients with nasophanyngeal carcinoma.^1^, ^2^ The ability to deliver complex dose distributions has allowed the delivery of simultaneous integrated boosts (SIB) to gross tumor volumes (GTV) alongside lower dose levels to at‐risk nodal regions.^(3)^ With intensity‐modulated plans it has been possible to reduce doses to nearby organs at risk (OAR). It has been shown that xerostomia rates in nasopharynx patients can be significantly reduced by lowering the mean parotid doses using IMRT.^(4)^ Conversely, studies have indicated that IMRT (accompanied by advances in functional imaging) may be effective in improving the therapeutic ratio by escalating the dose to the tumor.^5^, ^6^, ^7^ The concept of isotoxic planning — escalating the prescription dose until limiting organ at risk tolerances are met — has been demonstrated in a variety of clinical sites, including head and neck.^(8)^ Such a technique is reliant on the ability to deliver highly complex modulated treatment fields.

In many centers, the provision of IMRT has been accompanied (and in some cases supplanted) by the availability of volumetric‐modulated arc radiotherapy (VMAT). VMAT allows intensity‐modulated dose distributions to be delivered by rotating the linear accelerator (linac) around the patient while dynamically varying the gantry speed, dose delivery rate, and multileaf collimator (MLC) positions.^(9)^ This delivery method results in a much faster treatment time, and is often accompanied by a lower number of monitor units when compared to sliding window delivery of IMRT fields.^10^, ^11^, ^12^ Several treatment planning studies have compared VMAT to IMRT for head and neck treatments,^13^, ^14^, ^15^, ^16^ including nasopharyngeal carcinoma.^17^, ^18^, ^19^ Generally, VMAT has been found to provide similar target coverage to seven‐to nine‐field IMRT, while maintaining an acceptable or lower dose to nearby organs at risk. Mean parotid doses have also been shown to be similar or slightly lower with VMAT.^17^, ^19^

When moving to a new technique such as VMAT, planning studies are essential to determine any dosimetric differences to the established technique (in this case IMRT). However, there are a number of inherent problems and difficulties with computerized treatment planning studies. Foremost amongst these is the influence of the planner. VMAT, like IMRT, involves the selection of constraints for inverse optimization and an appropriate selection of constraints is essential to produce a plan which meets all of the treatment objectives. Many planning studies do not account for the possibility that the planner experience with each treatment technique may not be the same. Furthermore, there is the question of whether these studies are subject to expectation bias — that is, unblinded treatment planning may lead to users inadvertently biasing their plans in favor of the new technique. Some planning studies attempt to reduce user bias by using identical optimization constraints for both techniques (in this case IMRT and VMAT). However, the optimization algorithms themselves may be quite different,^20^, ^21^ such that this may not allow a fair comparison.

Ideally, comparative planning studies should involve a user‐independent selection of treatment planning parameters, with the plan aims clearly defined by the clinician from the outset. Automating the choice of optimization constraints would thus eliminate user‐dependence of the results, and allow for a fair comparison between treatment methodologies. Automated decision‐making techniques have been demonstrated previously for radiotherapy, such as the use of artificial neural networks to determine appropriate beam orientations.^(22)^ More recently, automated algorithms have been proposed for multicriteria optimization of IMRT,^23^, ^24^ and techniques have also been demonstrated for the automatic selection of IMRT optimization structures.^(25)^ However, such systems have not yet been applied to perform objective technique comparison studies.

The aim of this study is to compare dual‐arc VMAT to seven‐field IMRT for different nasopharynx treatment strategies. Firstly, the ability of each technique to produce a plan which meets the clinical objectives is investigated. Secondly, the ability to reduce dose to the parotid glands is assessed, while maintaining all other planning objectives. Finally, the ability of VMAT and IMRT to escalate dose to the tumor bed is investigated, keeping OAR doses within a tolerated range. In order to ensure a bias‐free comparison between the techniques, a novel automated planning method has been developed which requires a set of dosimetric aims and planning rules, defined a priori and identical for both planning methods. This system works within the environment of a commercial treatment planning system, such that the planning system's own direct aperture optimization is provided with a set of automatically generated optimization constraints. The comparison is made over ten nasopharynx patients, with the aim of objectively quantifying any benefit from rotational radiotherapy.

## II. MATERIALS AND METHODS

### A. Patients and treatment protocol

Ten nasopharyngeal carcinoma patients who had been previously treated with IMRT were randomly selected for this study. The median age at diagnosis was 55 (range 27‐64), with two patients originally presenting with stage I, four with stage II, and four with stage III disease. The standard treatment protocol employed was a three dose‐level prescription delivered in 33 fractions using a simultaneous integrated boost (SIB). The primary clinical target volume (CTV1) received 70 Gy, the high‐risk lymphatic nodes (CTV2) were treated with 63 Gy, and CTV3, representing the lower risk lymphatic involvement, received 56 Gy. Planning target volumes (PTVs) were created by adding a uniform margin of 3 mm around each CTV. All plan objectives, including maximum doses to organs at risk (OARs), are set out in Table 1.

The treatment planning system used was Pinnacle 9.0 (Philips Medical Systems, Madison, WI). Table 2 shows the beam arrangements and planning parameters employed, which remain the same for all patients. For the IMRT plans, a standard protocol using seven equispaced coplanar beams were set, with the treatment isocenter in the center of CTV1. The linac used for planning was an Elekta Synergy (Elekta, Stockholm, Sweden) with a 1 cm MLCi head for step‐and‐shoot IMRT delivery. Pinnacle's direct machine parameter optimization (DMPO) method was used.^(26)^ To allow for a high complexity of treatment plan, the maximum number of control points (i.e., MLC segments) was set to 100, the minimum segment area was set to 4cm^2^, and the minimum segment MUs was set to 2.

**Table 1:** Nasophanrynx target and OAR evaluation objectives. The spinal cord and brainstem tolerances are given for the planning reference volume (PRV), which includes a margin of 0.5 cm around the OAR

*Volume*	*Objective(s)*
*Level 1*	
PTV1	95%volume>95% prescription dose (66.5 Gy)
	99%volume>90% prescription dose (63 Gy)
PTV2	95%volume>95% prescription dose (59.9 Gy)
	99%volume>90% prescription dose (56.7 Gy)
PTV3	95%volume>95% prescription dose (53.2 Gy)
	99%volume>90% prescription dose (50.4 Gy)
Whole Body	Maximum 77 Gy
*Level 2*	
Spinal Cord PRV	Maximum 50 Gy
Brainstem PRV	Maximum 60 Gy
Optic Chiasm and Optic Nerves	Maximum 55 Gy
*Level 3*	
Cochleae	Maximum mean 40 Gy (target<35Gy)
Parotids	Maximum mean 26 Gy
Larynx	Maximum mean 50 Gy (target<45Gy)
Oral Cavity	Maximum mean 60 Gy (target<55Gy)
Eyes	Maximum 45 Gy (target<40Gy)

**Table 2:** Planning parameters used for optimization. The IMRT beam arrangement is currently employed clinically in our center for nasopharynx plans

*IMRT*	*VMAT*
7 co‐planar beams (gantry angles 205°, 255°, 305°, 0°, 50°, 105°, 155°)	2 arcs (clockwise and anticlockwise, 182° to 178°)
Minimum segment area 4 cm^2^	4° control point spacing
Minimum MU per segment 2	Maximum delivery time 300 s
Maximum number of segments 100	Leaf motion unconstrained between control points
Final dose calculation: Adaptive collapsed cone convolution	Final dose calculation: Adaptive collapsed cone convolution

Pinnacle's SmartArc optimization module was used to produce the VMAT plans.^(21)^ A dual arc strategy was employed, with the gantry rotating from 182° to 178° and vice versa. The collimator angle for each arc was set to 10° in order to reduce the cumulative contribution of interleaf leakage. A control point spacing of 4° was used, such that 90 control points per arc were available for optimization. The aim of this study was to compare the ability of IMRT and VMAT to produce highly complex treatment plans, neglecting any potential delivery time benefit with VMAT. As such, the constraints within SmartArc which aim to improve delivery efficiency (for example, maximum treatment time and maximum leaf motion per gantry degree) were relaxed so as to have a low bearing on the optimization (Table 2).

### B. Automated planning tool

**Figure 1:**
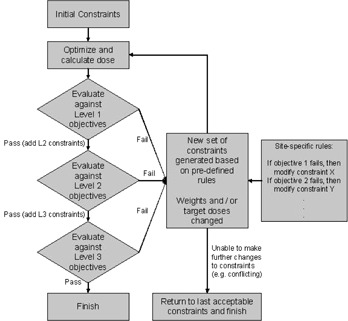
Flowchart describing the automated planning process employed.

A software tool has been developed which automatically adds and modifies optimization constraints based on the progress of the plan, through regular comparison to the plan objectives. The software follows a process shown in Fig. 1. Broadly, the planning system operations (such as optimization, saving the plan, represcribing the dose) were automated using Pinnacle scripts, whereas the evaluation of the plan objectives and creation of new constraints was performed by a Java application running alongside the scripts.

In principle, the system works by dividing the plan objectives into three different levels of priority, as labeled in Table 1. Initially, a set of optimization constraints are added which only aim to cover the target volumes (level 1). The plan is then inverse‐optimized (using DMPO or SmartArc, as described above) and the dose is calculated. Within Pinnacle, the current set of optimization constraints is then replaced by the evaluation objectives (Table 1). The evaluation objectives are a set of parameters that, if met, would likely result in a clinically acceptable plan. Optimization constraints, on the other hand, are used to drive the optimization such that the clinical requirements are met, and are generally not the same as the evaluation objectives. By recalculating the cost function of each evaluation objective, it is possible to determine which are passing or failing. If any evaluation objectives are not met at the current level, the software follows a set of predefined rules to generate a new set of optimization constraints.

The predefined rules are described by a separate initialization file, containing instructions as to which constraints need to change when a certain objective is not met, and how to change them. These rules are defined a priori for a given clinical site, and they aim to approximate the decision‐making processes during treatment planning. The rules may be simple — for example, if the minimum PTV1 objective is not being met (99% of the volume should receive at least 63 Gy), then the corresponding optimization constraint for PTV1 will be adjusted. Firstly, the adjustments are made by increasing the constraint weighting. All constraint weightings are initially set to ‘1', and if a change is required the weighting can be set to values of 10, 25, 50, 75, and then 100 sequentially. If, after reoptimization, the objective is still not met and the constraint weighting is set to the maximum value of 100, then the dose constraint can then be increased in 1 Gy steps (e.g., 64 Gy, 65 Gy, 66 Gy, ...) until the PTV1 coverage is achieved. Each objective in Table 1 is associated with at least one optimization constraint via these rules, and the rules are identical for all patients using both VMAT and IMRT. During the automated process, modifications to the optimization constraints continue iteratively until all of the evaluation objectives are met for level 1.

Once the target coverage level is passed, a second set of constraints are added to optimize serial‐like organs at risk (level 2). Again, the plan is optimized and then compared to the evaluation objectives to determine any failures. This time, if any of the level 1 objectives fail, then those constraint modifications are made ahead of any failures in level 2. This ensures that the higher priority objectives are always worked on ahead of lower importance objectives. This cycle of optimization, evaluation, and constraint modification is repeated until all of the level 1 and 2 objectives are passed.

**Figure 2:**
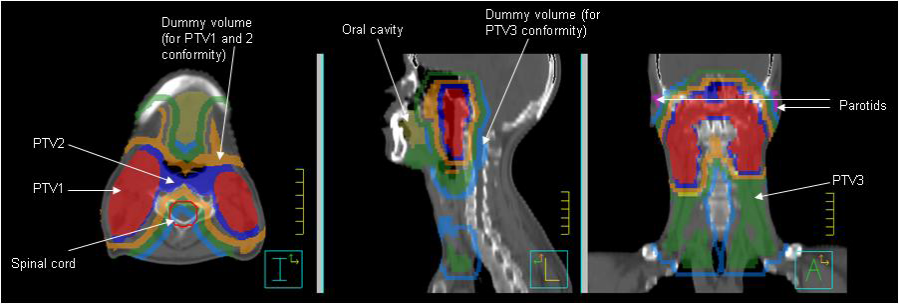
Typical regions of interest for one of the nasopharynx patients. Dummy structures were used alongside target volumes and organs at risk to aid optimization.

Following this, level 3 constraints are added, which consist of mainly parallel OARs where a lower dose is preferred, provided it is not at the expense of coverage to the targets (level 1) or exceeding the tolerance of serial OARs (level 2). Also included at this stage are dummy optimization structures, as shown in Fig. 2. There are three dummy structures, consisting of 1cm thick shells around each of the PTVs, which are applied to constrain the 95% isodose line of each PTV prescription. The purpose of these structures is to aid the conformity and homogeneity of dose to the target volumes. Within level 3, more complex predefined rules can be applied. For example, if it is observed that the target and serial OAR objectives are being satisfied, then all of the dummy structure constraints can be tightened, thus aiming to improve the conformity of the whole dose distribution. Ultimately, the software will continue making adjustments until one or more of the constraints can no longer be adjusted (for example, if it conflicts with another constraint). At this stage, the software ‘rolls back’ to the last set of constraints that met all of the plan evaluation objectives. The plan is only considered complete if all of the objectives are met.

### C. Experimental treatment strategies

The automated planning method was used to investigate three treatment strategies. Firstly, the ability of each technique to produce a standard clinically acceptable plan was investigated. The objectives as set out in Table 1 were used in this instance. The automated planning system followed the set of rules detailed above (i.e., target coverage, then serial OAR avoidance, followed by parallel OAR reduction as far as possible).

Secondly, a parotid‐sparing strategy was investigated. For the purposes of this experiment, all other level 3 OARs were provided with tolerance doses which, once reached, were considered acceptable and no longer optimized. For example, the target mean cochlea dose was set to 35 Gy in the evaluation objectives. For the mean parotid OAR, no such target dose was set, so the system lowered the parotid dose until a higher level objective irreversibly failed.

The final treatment strategy to be investigated was the ability to escalate the prescription dose to the primary target volume (CTV1). In this case, the automated planning system maintained the level 2 and 3 OAR doses below the maximally tolerated levels set out in Table 1. Whenever all objectives were met, however, the system escalated the prescription (to the mean of CTV1) by 2 Gy. The process of optimization and dose escalation continued until one of the OAR doses exceed their tolerance, and it was not able to make further changes to the constraint parameters. The prescription dose at this stage is then taken as the limiting prescription dose for that patient.

All strategies were applied over the ten patients using both IMRT and VMAT. The modification rules and evaluation objectives were the same for both delivery methods. As the entire process was automated, there was no requirement for the planning to be supervised or interrupted. The total number of optimization steps was recorded along with the total planning time for each patient. Dose‐volume parameters were then retrieved for comparison between the IMRT and VMAT plans. All comparisons were made using a nonparametric Wilcoxon signed‐rank test, where statistical significance was taken if p<0.05. Where applicable mean values have been quoted with 1 standard deviation in parentheses.

## III. RESULTS

### A. Standard planning

For standard planning, the automated tool produced plans which met the objectives in all of the patients, using both VMAT and IMRT. The number of steps required for the automated system to produce an acceptable VMAT plan was lower than that for IMRT (mean 36 steps compared to 50 for IMRT, p<0.05). While the number of steps was lower for VMAT, the total planning time was significantly longer at 7.0 hours compared to 1.8 hours for IMRT (p<0.01), which was due to the increased time per SmartArc optimization. An example set of final optimization constraints are provided in Table 3.

**Figure 3:**
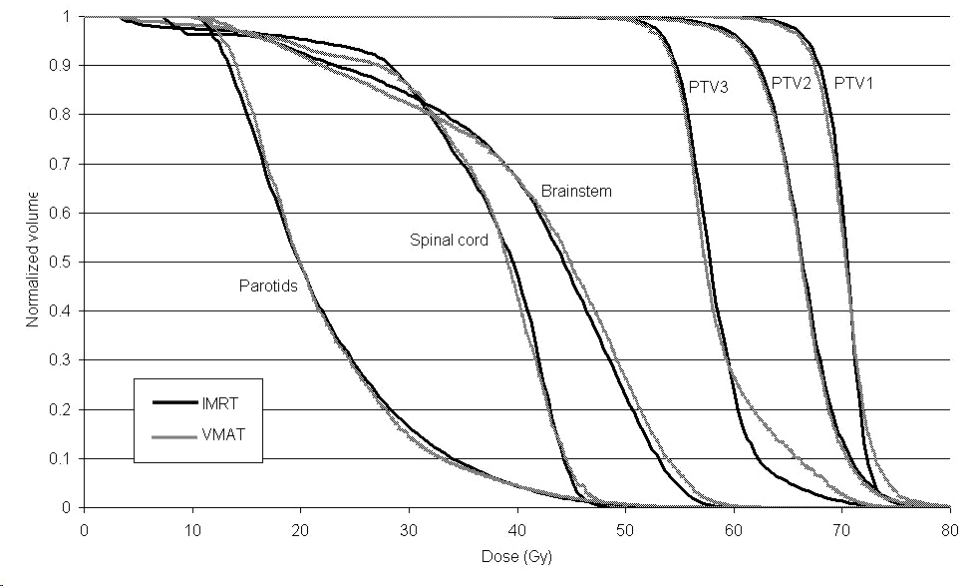
Averaged dose‐volume histogram over all ten nasopharynx patients, comparing the VMAT and IMRT automatically generated solutions.

The number of monitor units for the IMRT plans was 731.8±62.5MU compared to 642.2±51.6MU for the VMAT plans. For all ten plans with both delivery techniques, the stopping point for the automated system was when the minimum PTV1 constraint exceeded the uniform dose constraint, resulting in a conflict and hence a rolling back to previously acceptable values. Figure 3 shows an averaged dose‐volume histogram comparing VMAT and IMRT. Dosimetric results are given in Table 4. Heterogeneity index, defined as the ratio of the dose received by 5% and 95% of the volume, is also reported for each PTV. For all the target volumes, level 2 and level 3 OARs, no significant differences were identified between VMAT and IMRT (p>0.2 for all objectives).

**Table 3:** The final constraints for one of the nasopharynx IMRT plans, as produced by the automated system. This set of constraint weightings and doses produced a final plan which met all of the dose‐volume objectives

*Constraints*	*Constraint Dose (Gy)*	*Weighting*
*Level 1*		
PTV1 minimum dose to 95% volume	66.5	75
PTV1 minimum dose	63.0	1
PTV2 minimum dose to 95% volume	62.9	100
PTV2 minimum dose	61.7	100
PTV3 minimum dose to 95% volume	55.2	100
PTV3 minimum dose	57.4	100
Whole Body maximum dose	72.0	100
PTV1 uniform dose	70.0	25
*Level 2*		
Spinal Cord PRV maximum dose	45.0	100
Brainstem PRV maximum dose	60.0	50
Optics maximum dose	50.0	50
*Level 3*		
Cochlea maximum EUD	40.0	1
Parotid maximum EUD	16.0	100
Larynx maximum EUD	50.0	1
Oral Cavity maximum EUD	60.0	25
Eyes maximum dose	45.0	1
PTV1 limiting rind maximum dose	66.5	100
PTV2 limiting rind maximum dose	59.9	100
PTV3 limiting rind maximum dose	50.4	75

**Table 4:** Dosimetric results for the standard VMAT and IMRT planning, averaged over the ten patients. Standard deviation is shown within parentheses

*Volume*		*IMRT*	*VMAT*
PTV1	V95%	95.8%±1.3%	97.1%±2.3%
	V90%	99.3%±0.4%	99.4%±0.2%
	HI (D95/D5)	1.09±0.03	1.12±0.02
PTV2	V95%	96.9%±0.5%	97.6%±1.4%
	V90%	99.0%±0.3%	99.2%±0.7%
	HI (D95/D5)	1.19(±0.05	1.17±0.03
PTV3	V95%	96.8%±1.2%	97.4%±1.8%
	V90%	99.2%±0.4%	99.4%±0.6%
	HI (D95/D5)	1.21±0.09	1.16±0.08
Spinal cord PRV		46.8±0.7Gy	47.4±1.3Gy
Brainstem PRV		56.6±1.1Gy	56.9±3.6Gy
Optic chiasm and optic nerves		45.5±5.6Gy	45.7±3.8Gy
Cochleae		39.3±1.6Gy	37.1±2.9Gy
Parotids		25.9±3.2Gy	26.8±1.5Gy
Larynx		48.3±1.2Gy	45.6±4.3Gy
Oral cavity		50.7±1.6Gy	48.9±8.7Gy
Eyes		29.7±11.9Gy	31.2±9.7Gy

### B. Parotid sparing

**Figure 4:**
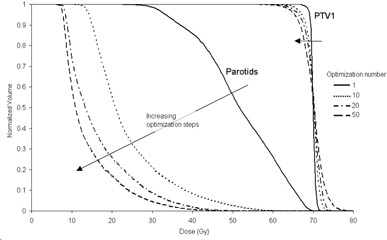
Example of the progress of the dose‐volume histograms over the course of the parotid dose optimization.

With the automated system adjusted to concentrate only on lowering the mean parotid dose, VMAT was found to be capable of a greater reduction than IMRT. All other objectives remained within the acceptable tolerances. The minimum parotid doses for each patient are displayed in Table 5. For the IMRT patients, the planning tool reduced the mean parotid dose to 19.6 Gy over the ten patients (range 13.9‐25.0 Gy). For the VMAT plans this figure was 17.0 Gy (13.1‐23.8 Gy). The biggest reduction was observed in patient 9, whose mean parotid dose was reduced from 19.0 Gy with IMRT to 13.1 Gy with VMAT, a difference of 5.9 Gy. On average, the mean parotid dose was reduced by 2.5 Gy using VMAT compared to IMRT (p<0.01). Figure 4 demonstrates how the dose‐volume histogram changes over the automated planning process. For these plans, the mean number of MUs was 800.6±87.0 for IMRT and 665.9±72.1 for VMAT. No correlation was observed between tumor staging (or extent of neck nodes) and the ability to reduce parotid dose.

**Table 5:** Lowest parotid doses achieved using IMRT and VMAT, while maintaining all other plan objectives. TNM stages are given for each patient

		*Mean parotid dose (Gy)*		
	*Stage*	*IMRT VMAT*	Δ(IMRT−VMAT)
P1	$T_{3}N_{2}$	19.4	19.1	0.3
P2	$T_{1}N_{2}$	18.3	16.7	1.6
P3	$T_{2}N_{3}$ ^a^	13.9	13.8	0.1
P4	$T_{2}N_{1}$	25.0	23.8	1.2
P5	$T_{2}N_{1}$ ^a^	20.6	15.5	5.0
P6	$T_{3}N_{1}$ ^a^	18.8	17.2	1.6
P7	$T_{2}N_{2}$ ^a^	23.4	21.5	1.9
P8	$T_{3}N_{2}$	20.5	15.6	4.9
P9	$T_{2}N_{1}$	19.1	13.1	5.9
P10	$T_{3}N_{1}$ ^a^	16.7	13.9	2.7
Average		19.6	17.0	2.5

### C. Dose escalation

For the third strategy under investigation, dose escalation, all OARs were kept within their maximally tolerable doses set out in Table 1. The limiting prescription doses (prescribed to the mean of CTV1) are given for each patient in Table 6. On average, the system was able to escalate the dose to 91.6±8.2Gy for IMRT patients and 90.8±5.8Gy for VMAT patients. No statistically significant difference was observed between the ability of the two treatment techniques to escalate to a maximum limiting dose (p>0.5 over the ten patients). Again, no correlation was observed between the maximum prescription doses and the tumor staging or the location of nodal involvement, but it was observed that patients with predominantly unilateral neck nodes had larger absolute differences between the IMRT and VMAT escalation doses (average difference of 10.8±5.0Gy vs. 2.0±2.4Gy for bilateral nodes). The stopping point for the automated system varied between patients. The most common limiting objective was the PTV2 prescription (five patients), followed by the brain stem maximum dose (three patients) and the spinal cord maximum dose (two patients). These limiting objectives were the same using both the IMRT and VMAT planning techniques.

**Table 6:** Highest prescription dose achieved (using 2 Gy steps from the standard prescription of 70 Gy) to the mean of CTV1, maintaining all other objectives and OAR doses within tolerance. TNM stages are given for each patient

		*Prescription Dose (mean to CTV1 in Gy)*		
	*Stage*	*IMRT*	*VMAT*	Δ(IMRT−VMAT)
P1	$T_{3}N_{2}$	94.0	96.0	−2.0
P2	$T_{1}N_{2}$	88.0	86.0	2.0
P3	$T_{2}N_{3}$ ^a^	102.0	84.0	18.0
P4	$T_{2}N_{1}$	82.0	88.0	−6.0
P5	$T_{1}N_{1}$ ^a^	86.0	96.0	−10.0
P6	$T_{3}N_{1}$ ^a^	76.0	86.0	−10.0
P7	$T_{2}N_{2}$ ^a^	88.0	84.0	4.0
P8	$T_{3}N_{2}$	92.0	92.0	0.0
P9	$T_{2}N_{1}$	98.0	98.0	0.0
P10	$T_{3}N_{1}$ ^a^	110.0	98.0	12.0
Average		91.6	90.8	0.8

## IV. DISCUSSION

The value of arc radiotherapy in the clinic remains a popular topic of research, with many publications investigating the similarities and differences to static beam IMRT.^10^, ^11^, ^13^, ^14^, ^15^, ^16^ Many of these comparative studies demonstrate a significant improvement with VMAT in terms of the monitor unit efficiency and delivery time. If the delivery benefit is disregarded, however, it is more difficult to identify situations in which VMAT offers a dosimetric treatment benefit over IMRT. In this study, by attempting to reduce planner bias and by reducing the influence of VMAT's delivery constraints, it has been possible to more objectively compare these two treatment paradigms.

For standard planning, the automated tool was able to produce plans which met the clinical objectives in all of the patients. All plans were also assessed by experienced IMRT and VMAT planners, and found to be clinically acceptable. As the system was provided with the same set of decision rules for both VMAT and IMRT, it is expected that the target coverage and serial OAR doses are similar for both techniques (the automated tool is designed to take these values to close to their tolerance). However, when the parotid doses were optimized, the VMAT plans were able to generate a significantly lower mean dose before one of the higher‐level objectives failed. The difference between the VMAT and IMRT mean parotid doses was 2.5 Gy on average. The QUANTEC project reviewed several dose‐response studies for xerostomia and found that, for studies with long‐term follow up (>12months), the reduction in stimulated salivary flow rate was approximately 1.5% for every 1 Gy of mean dose received by the parotids.^(27)^ Based on this, a reduction of 2.5 Gy could represent a 3.75% improvement in long‐term salivary flow rate. It should be stressed, however, that this is highly patient‐specific and will be influenced by other factors such as baseline function.

These results agree with previous comparisons between IMRT and VMAT which have shown parotid doses to be either equivalent^11^, ^15^ or slightly lower with VMAT.^10^, ^13^, ^16^, ^17^, ^18^ Other planning studies also reported marginal improvements in target coverage with VMAT, although this was not observed in this study. One of the main differences between this planning study and others reported in the literature is the use of a user‐free system. The purpose of this was to ensure that the planning was independent of planner experience with both techniques. Some planning comparison studies have previously attempted to address user bias by using identical optimization constraints for both the techniques being investigated.^(28)^ The use of identical optimization constraints, however, may not produce a fair comparison if the optimization algorithm itself is significantly different (as with the Pinnacle system).

While VMAT was found to provide a potential benefit for the reduction of parotid doses, no such benefit was determined for a dose escalation strategy. The automated system was able to produce plans which allowed substantive simultaneous boosts to the primary target volume (up to 110 Gy in one patient). However, the limiting prescriptions between the IMRT plans and VMAT plans showed significant variation — differences of up to 18 Gy between each technique. There were no trends suggesting whether IMRT or VMAT is a more suitable technique for dose escalation. One conclusion which can be drawn from this is that interpatient variability is larger than the difference between IMRT and VMAT planning.

It is of interest to observe that patients with predominantly unilateral neck nodes elicited larger differences between the IMRT and VMAT plans than those patients with bilateral disease. While again there was no technique superior for these patients, it indicates that geometry of the target volumes has some influence over the choice of treatment strategy. Work is now underway to determine whether the anatomical characteristics of the patient (such as position of neck nodes, or the proximity of target volumes to nearby OARs) can be used to predict whether VMAT or IMRT is a better candidate for dose escalation. It should be noted that the clinical benefit of such dose escalation is beyond the scope of this paper. Consideration will need to be given to OARs other than those in Table 1. OARs such as the mandible, submandibular glands, temporal lobes, temporomandibular joint, and brachial plexus may further limit the achievable escalated prescription dose.

While the automated planning tool described here can produce acceptable plans in Pinnacle unsupervised, there remain some limitations. Only the optimization constraints were automatically generated and, as such, this system still required a planner‐based choice of beam parameters (e.g., number and orientation of beams, number of arcs, collimator rotation and control point spacing). It may be appropriate instead to consider the automation of patient‐specific beam orientations for IMRT. For the purposes of this comparison study, the beam parameters as shown in Table 2 were used, as they are similar to clinical practice. The automated system as described here has not been applied for clinical plan production. Larger scale benchmarking against manually produced plans would be required prior to any clinical implementation. Presently, the intention is to use this system to investigate automated isotoxic planning (i.e., to generate individualized, dose‐escalated plans). Further anticipated applications of the automated system include a) treatment technique comparisons, b) benchmarking of new planning software, or c) development of class solutions for new clinical sites.

We anticipate that the planning strategy adopted here can be generalized for other treatment scenarios. With many treatments, it should be possible to generate a set of plan objectives as in Table 1, and then divide them into priority‐based levels of target coverage, serial organ avoidance, and parallel organ dose reduction (or general dose conformity). The technique here has involved associating each objective with a rule which states that, if that objective is not being met, then certain optimization constraints must be changed, and changes are prioritized based on the ‘level’ of the failed objective. While we have automated this strategy through the use of scripts and a Java application, it is possible in principle to perform these steps manually. This may be useful, for example, when attempting to generate ‘off‐protocol’ or infrequent treatment plans, for sites which do not have a class solution.

The use of a set of rules to ‘search’ for appropriate optimization parameters within a commercial planning system can be contrasted with other systems. In particular, the iCycle algorithm is an independent optimization system, guided by priority‐assigned clinical objectives.^(23)^ This system allows for a wide range of parameters to be optimized (including beam and couch orientation), potentially making it a candidate for bias‐free plan comparison studies. Another automated option has been proposed by Janssen et al.^(29)^ This system creates a large number of IMRT and VMAT plans using a commercial planning system with a range of optimization constraints, forming a pareto front for a given set of objectives. By producing pareto fronts for both techniques, it is possible to determine which technique is optimal by comparing the fronts. As this system requires many hundreds of optimizations, the plans take a number of days to produce. Other studies have demonstrated the use of unsupervised learning systems for the creation of treatment plans,^30^, ^31^ determining IMRT beam angles,^(22)^ and identifying optimum patient position.^(32)^ However, we have been unable to find previous studies demonstrating the use of a planner‐free system to compare two treatment techniques (VMAT and IMRT) within the environment of a commercial planning system.

## V. CONCLUSIONS

An automated planning tool has been developed to perform a comparison study between seven‐field IMRT and dual‐arc VMAT. The system was able to generate clinically acceptable plans with both treatment techniques and, when given instructions to reduce mean parotid doses as far as possible, it was found that the VMAT plans were capable of a significantly lower mean parotid dose compared to IMRT. This study indicates that VMAT offers a parotid‐sparing benefit over IMRT in the treatment of nasopharyngeal carcinoma, which could lead to reduced xerostomia rates. Conversely, investigating a strategy of dose escalation to the primary target volume, VMAT and IMRT gave a large range of maximally tolerated doses, with no technique superior over all ten patients.

## ACKNOWLEDGMENTS

This work was partly funded by an Elekta research grant.
